# Adolescent Victimization and Self-Injurious Thoughts and Behaviors: A Genetically Sensitive Cohort Study

**DOI:** 10.1016/j.jaac.2018.07.903

**Published:** 2019-05

**Authors:** Jessie R. Baldwin, Louise Arseneault, Avshalom Caspi, Terrie E. Moffitt, Helen L. Fisher, Candice L. Odgers, Antony Ambler, Renate M. Houts, Timothy Matthews, Dennis Ougrin, Leah S. Richmond-Rakerd, Ryu Takizawa, Andrea Danese

**Affiliations:** a King’s College London, UK; b National and Specialist CAMHS Clinic for Trauma, Anxiety, and Depression, South London and the Maudsley NHS Foundation Trust, London, UK; c Duke University, Durham, NC; d University of California, Irvine; e Duke University; f Graduate School of Education, The University of Tokyo, Japan

**Keywords:** victimization, suicidal ideation, self-harm, suicide attempt, adolescence

## Abstract

**Objective:**

Victimized adolescents have an increased risk of self-injurious thoughts and behaviors. However, poor understanding of causal and non-causal mechanisms underlying this observed risk limits the development of interventions to prevent premature death in adolescents. This study tested whether pre-existing family-wide and individual vulnerabilities account for victimized adolescents’ increased risk of self-injurious thoughts and behaviors.

**Method:**

Participants were 2,232 British children followed from birth to 18 years of age as part of the Environmental Risk Longitudinal Twin Study. Adolescent victimization (maltreatment, neglect, sexual victimization, family violence, peer/sibling victimization, cyber victimization, and crime victimization) was assessed through interviews with participants and co-informant questionnaires at the 18-year assessment. Suicidal ideation, self-harm, and suicide attempt in adolescence were assessed through interviews with participants at 18 years.

**Results:**

Victimized adolescents had an increased risk of suicidal ideation (odds ratio [OR] 2.40, 95% CI 2.11–2.74), self-harm (OR 2.38, 95% CI 2.10–2.69), and suicide attempt (OR 3.14, 95% CI 2.54–3.88). Co-twin control and propensity score matching analyses showed that these associations were largely accounted for by pre-existing familial and individual vulnerabilities, respectively. Over and above their prior vulnerabilities, victimized adolescents still showed a modest increase in risk for suicidal ideation (OR 1.45, 95%CI 1.10–1.91) and self-harm (OR 1.50, 95% CI 1.18–1.91) but not for suicide attempt (OR 1.28, 95% CI 0.83–1.98).

**Conclusion:**

Risk for self-injurious thoughts and behaviors in victimized adolescents is explained only in part by the experience of victimization. Pre-existing vulnerabilities account for a large proportion of the risk. Therefore, effective interventions to prevent premature death in victimized adolescents should not only target the experience of victimization but also address pre-existing vulnerabilities.

Suicide is the third leading cause of death in adolescents worldwide.^1^ Suicide attempts are often preceded by suicidal ideation and self-harm,^2^ which are particularly prevalent in adolescents.^3^ To prevent self-injurious thoughts and behaviors during adolescence, it is important to identify proximal risk factors that can be modified through intervention.^4^, ^5^

The role of adolescent victimization is considered in this study. One in 3 adolescents experience severe victimization^6^ from exposures in the community (eg, crime, sexual victimization, and bullying) and in the family (eg, maltreatment).^7^, ^8^, ^9^ These stressful experiences may be particularly harmful to adolescents because of the major neurobiological, emotional, and social changes that take place during this period.^10^, ^11^ Previous studies have suggested that victimized adolescents have an increased risk for self-injurious thoughts and behaviors.^12^, ^13^, ^14^, ^15^ However, confusion about the relative contribution of causal and non-causal mechanisms complicates the interpretation of these findings and hampers the development of effective interventions.^16^

Victimized adolescents might be at high risk for self-injurious thoughts and behaviors by virtue of exposure to maltreatment, bullying, or crime. Alternatively, their risk might be high due to pre-existing liability and experiences. This alternative non-causal interpretation is plausible because family-wide factors (eg, family history of psychopathology, socioeconomic disadvantage) and individual factors (eg, childhood victimization, cognitive deficits, stress-reactive personality traits) can predispose adolescents to experience victimization^6^, ^17^, ^18^ and influence their risk for self-injurious thoughts and behaviors.^19^

In this study, we carried out a stringent test of these non-causal interpretations, capitalizing on design and analytical features with complementary strengths. To account for family-wide factors, we used a co-twin control design^20^ to test whether adolescents with the same genotype and rearing environment—but different exposure to adolescent victimization—had a different risk for self-injurious thoughts and behaviors. To account for individual factors, we used propensity score matching^21^ to test whether adolescents with a similar individual propensity to experience victimization—but different exposure to adolescent victimization—had a different risk for self-injurious thoughts and behaviors.

## Method

### Study Sample

Participants were members of the Environmental Risk (E-Risk) Longitudinal Twin Study, which tracks the development of a birth cohort of 2,232 British children. The sample was drawn from a larger birth register of twins born in England and Wales in 1994 to 1995.^22^ Full details about the sample are reported elsewhere.^23^ Briefly, the E-Risk sample was constructed in 1999 to 2000, when 1,116 families (93% of those eligible) with same-sex 5-year-old twins participated in home-visit assessments. This sample was composed of 56% monozygotic and 44% dizygotic twin pairs; sex was evenly distributed within zygosity (49% male). Families were recruited to represent the UK population of families with newborns in the 1990s based on residential location throughout England and Wales and mother’s age. Teenage mothers with twins were over-selected to replace high-risk families who were selectively lost to the register through non-response. Older mothers having twins by assisted reproduction were under-selected to avoid an excess of well-educated older mothers. The study sample represents the full range of socioeconomic conditions in Great Britain, as reflected in the families’ distribution in a neighborhood-level socioeconomic index (A Classification of Residential Neighbourhoods [ACORN], developed by CACI Inc. [Arlington, VA] for commercial use in Great Britain)^24^: 25.6% of E-Risk families live in “wealthy achiever” neighborhoods compared with 25.3% nationwide; 5.3% versus 11.6% live in “urban prosperity” neighborhoods; 29.6% versus 26.9% live in “comfortably off” neighborhoods; 13.4% versus 13.9% live in “moderate means” neighborhoods; and 26.1% versus 20.7% live in “hard-pressed” neighborhoods. The E-Risk study under-represents “urban prosperity” neighborhoods because such households are likely to be childless.

Follow-up home visits were conducted when the children were 7 (98% participation), 10 (96%), 12 (96%), and 18 (93%) years old. Home visits at 5, 7, 10, and 12 years of age included assessments with participants and their mother (or primary caretaker); the home visit at 18 years included interviews only with participants. Each twin participant was assessed by a different interviewer. The average age of the twins at the time of the assessment was 18.4 years (standard deviation 0.36); all interviews were conducted after the 18th birthday. There were no differences between the 2,066 participants who took part at 18 years and those who did not in terms of socioeconomic status assessed when the cohort was initially defined (χ^2^ = 0.86, *p* = .65), IQ scores at 5 years (*t* = 0.98, *p* = .33), internalizing or externalizing behavior problems at 5 years (*t* = 0.40, *p* = .69 and *t* = 0.41, *p* = .68, respectively), or childhood victimization (*z* = 0.51, *p* = .61). Of the study members who participated in the assessment at 18 years, 99.5% (2,055) had complete data on adolescent victimization and self-injurious thoughts and behaviors.

The Joint South London and Maudsley and the Institute of Psychiatry Research Ethics Committee approved each phase of the study. Parents gave informed consent and twins gave assent at 5 to 12 years of age and then informed consent at 18 years.

### Adolescent Victimization

These measures have been described previously^6^ and details are provided in Supplement 1, available online. Briefly, at age 18 years, participants were interviewed about exposure to a range of adverse experiences between ages 12 to 18 years using the Juvenile Victimization Questionnaire Second Revision (JVQ-R2)^25^ adapted as a clinical interview. Each co-twin was interviewed by a different research worker, and each JVQ question was asked for the period “since you were 12.” Twelve years is a salient age for these participants because it is the age when British children leave primary school to enter secondary school. The JVQ has good psychometric properties^26^ and was used in the UK National Society for the Prevention of Cruelty to Children national survey,^27^, ^28^ thereby providing important benchmark values for comparisons with our cohort. Our adapted JVQ-R2 was composed of 45 questions covering 7 different forms of victimization: maltreatment, neglect, sexual victimization, family violence, peer/sibling victimization, cyber victimization, and crime victimization. Exposure to each type of adolescent victimization was coded by trained raters using a 3-point scale (0 = “no exposure”; 1 = “probable” or “less severe” exposure; 2 = “definite” or “severe” exposure).

The adolescent poly-victimization variable was derived by summing all victimization experiences that received a code of 2 (ie, severe exposure): 64.6% of adolescents had 0 severe victimization experiences; 19.2% had 1; 9.4% had 2; 4.5% had 3; 1.5% had 4; 0.5% had 5; and 0.2% had 6. The adolescent poly-victimization distribution was winsorized by combining 3, 4, 5, and 6 severe victimization experiences into 1 category (≥3 experiences), resulting in a 4-category poly-victimization variable (0, 1, 2, and ≥3 severe victimization experiences).

#### Informant Reports of Adolescent Victimization

At age 18 years, each study member’s co-twin and parent (usually their mother) were asked to reply to a confidential questionnaire that inquired whether the study member had ever been the victim of each of the 7 different forms of victimization assessed in the adapted JVQ-R2 interview. We summed affirmative responses to these questions, within each reporter. Correlations (*r*) were 0.38 between co-twin and parental reports, 0.38 between co-twin and study members’ JVQ reports, and 0.34 between parental and study members’ JVQ reports.

### Self-Injurious Thoughts and Behaviors

Study members were privately interviewed at age 18 years about suicidal ideation, self-harm, and suicide attempts since 12 years of age using a life history calendar. To assess suicidal ideation, participants were asked whether they had thought it would be better if they were dead or had thought about a plan to commit suicide. We defined suicidal ideation as an affirmative answer to either of these questions. To assess self-harm, participants were asked whether they had tried to hurt themselves to cope with stress or emotional pain. To assess suicide attempt, participants were asked whether they had tried to kill themselves. No study member completed suicide. Participants who reported self-harm or suicide attempt were further queried about the types of self-injurious behavior that they engaged in. Ten behaviors were probed (eg, cutting, burning, overdose), plus the option to describe any other way they had hurt themselves.

### Individual Factors Included in Propensity Score

To account for pre-existing individual differences between victimized and non-victimized adolescents, we derived a propensity score for adolescent victimization. The propensity score included 11 child-specific factors prospectively measured before 12 years of age and selected based on previous findings^6^, ^18^, ^29^, ^30^: childhood victimization, social isolation, IQ, internalizing problems, externalizing problems, self-harm, and traits composing the 5-factor model of personality (openness to experience, conscientiousness, extraversion, agreeableness, and neuroticism; for details, see Table S1, available online). Participants with missing data for these covariates (n = 119) did not differ from those with complete data (n = 1,936) according to adolescent victimization and self-injurious thoughts and behaviors (Table S2, available online).

### Statistical Analysis

We calculated prevalence rates, sex differences in prevalence, and heritability estimates for data on suicidal ideation, self-harm, and suicide attempt. Sex differences in outcomes were estimated using generalized estimating equations (GEEs) with binomial function and an exchangeable correlation structure to account for familial clustering in STATA 15 (StataCorp, College Station, TX). Heritability estimates were calculated using “Open Mx” in R (R Foundation, Vienna, Austria).

Next, we used GEE analyses to test the associations of adolescent poly-victimization with self-injurious thoughts and behaviors; the sensitivity of findings across informants to examine common-method bias^31^; and the sensitivity of findings across different measure components (7 individual victimization types).

To test whether family-wide factors confounded the associations, we used a co-twin control design with GEEs to parse the effect of adolescent poly-victimization on self-injurious thoughts and behaviors into *between-twin pair effects* and *within-twin pair effects.*^32^ The within-twin pair effects show whether a twin with higher poly-victimization has a greater risk of self-injurious thoughts and behaviors than their less victimized co-twin. Because twins share their rearing environment and half (dizygotic twins) or all (monozygotic twins) their segregating genes, significant within-twin pair effects would indicate that adolescent poly-victimization is associated with self-injurious thoughts and behaviors independent of the rearing environment and genetic influences (specifically, half the genetic influences in analyses of dizygotic twins or all genetic influences in analyses of monozygotic twins).

To test whether individual factors confounded the associations, we used 3 methods. First, we used multivariate GEE analyses to test whether any of the selected individual risk factors accounted for the association between adolescent victimization and self-injurious thoughts and behaviors. Second, we accounted for all of these individual risk factors simultaneously by using the STATA command “teffects psmatch” (with robust standard errors) to derive a propensity score for adolescent victimization (ie, exposure to 1, 2, or ≥3 victimization types) versus no victimization and used 1:1 nearest neighbor matching with replacement to match each study member to a study member with a similar propensity score in the opposite “treatment” group (eg, victimization [n = 671] or no victimization [n = 1,265]). As recommended,^33^ we used a caliper width of 0.2 of the standard deviation of the logit of the propensity score, which was sufficient to ensure that each study member was matched to a study member in the opposite treatment group. Then, we estimated the average treatment effect, which reflects the excess prevalence of self-injurious thoughts and behaviors in victimized adolescents versus non-victimized adolescents matched for the propensity score. Third, to estimate the joint bias owing to family-wide and individual factors, we expanded the monozygotic co-twin control regression model by also accounting for within-twin pair differences in the propensity score (ie, the extent to which twins in a pair differ on individual factors that predispose to victimization). This enabled us to test whether a twin exposed to higher poly-victimization was more likely to experience self-injurious thoughts and behaviors than their less victimized co-twin, once pre-existing individual vulnerabilities were accounted for.

Further details of the statistical analyses are provided in Supplement 2, available online.

## Results

### Self-Injurious Thoughts and Behaviors in Adolescence

**Figure 1:**
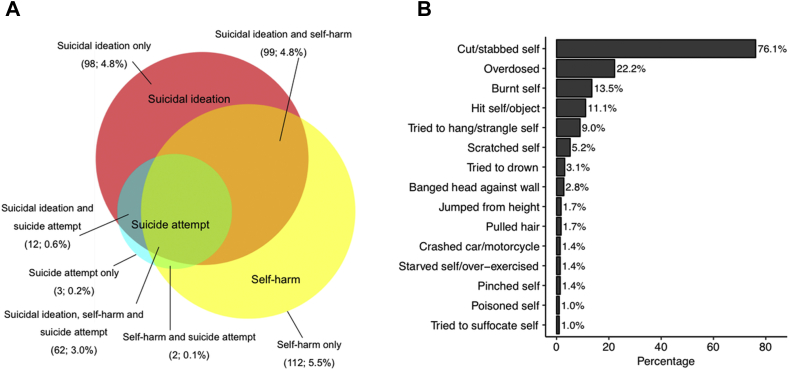
Distribution of Self-Injurious Thoughts and Behaviors in Adolescence ***Note:****(A) Overlap between adolescent suicidal ideation, self-harm, and suicide attempt. The size of the circles and their overlap is proportional to the number of participants (N = 2,055). Suicidal ideation was correlated with self-harm (r = 0.80,* p *< .001) and suicide attempt (r = 0.89,* p *< .001). Self-harm was correlated with suicide attempt (r = 0.79,* p *< .001). (B) Prevalence of self-injurious behaviors endorsed by more than 1% of those who reported self-harm or suicide attempt. Girls and boys did not differ in types of self-injury reported, except for cutting/stabbing self (more prevalent in girls; odds ratio 1.94,* p *= .021) and hitting self/object (less prevalent in girls; odds ratio 0.24,* p *< .001). Please note color figures are available online.*

Nearly a fifth (18.9%; n = 388) of study members reported some form of self-injurious thoughts and behaviors, with 13.2% (n = 271) reporting suicidal ideation, 13.4% (n = 275) reporting self-harm, and 3.8% (n = 79) reporting suicide attempt, with substantial overlap between groups (Figure 1A). Of those who reported self-harm or suicide attempt, cutting was the most prevalent self-injurious behavior (76.1%), followed by overdosing (22.2%) and burning (13.5%; Figure 1B). The overall prevalence of self-harm was greater in girls than in boys (odds ratio [OR] 1.79, 95% CI 1.34–2.39, *p* < .001), but there were no significant sex differences in the prevalence of suicidal ideation (OR 1.29, 95% CI 0.96–1.72, *p* = .09) or suicide attempt (OR 1.34, 95% CI 0.82–2.22, *p* = .25). The occurrence of self-injurious thoughts and behaviors was explained in part by genetic influences, with heritability estimates of 61% (95% CI 47%–72%) for suicidal ideation, 58% (95% CI 44%–70%) for self-harm, and 62% (95% CI 37%–80%) for suicide attempt (Figure S1, available online).

### Are Victimized Adolescents at Greater Risk for Self-Injurious Thoughts and Behaviors?

**Figure 2:**
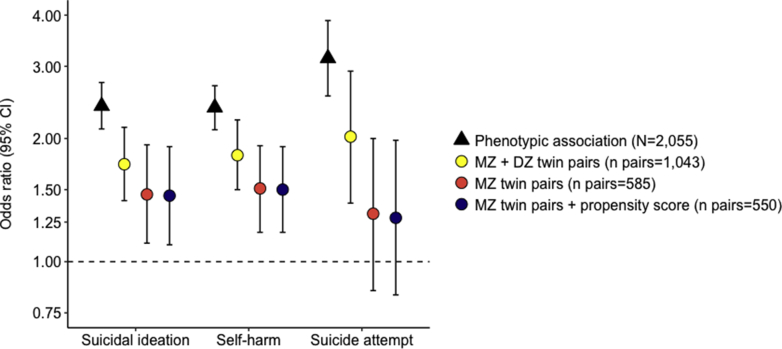
Association Between Adolescent Victimization and Self-Injurious Thoughts and Behaviors ***Note:****DZ = dizygotic; MZ = monozygotic. Please note color figures are available online.*

Adolescents reporting exposure to more victimization types were at greater risk for suicidal ideation (OR 2.40, 95% CI 2.11–2.74), self-harm (OR 2.38, 95% CI 2.10–2.69), and suicide attempts (OR 3.14, 95% CI 2.54–3.88) between ages 12 to 18 years (Table 1, model 1; black triangles in Figure 2). Risk estimates in victimized adolescents were similar in boys and girls (Table S3, available online); thus, we hereafter present analyses in the overall sample. In sensitivity analyses, we found that adolescents identified by their co-twin or parent as having been victimized also showed increased risk for self-injurious thoughts and behaviors (Table 1, models 2 and 3), suggesting that the findings were not due to biased self-reports of victimization by adolescents who experienced self-injurious thoughts and behaviors. Furthermore, adolescents reporting exposure to each of the 7 individual types of victimization showed greater risk for self-injurious thoughts and behaviors compared with unexposed adolescents (Table S4, available online).

**Table 1:** Association Between Adolescent Victimization and Self-Injurious Thoughts and Behaviors

	Model 1 (Self-Report of Victimization; N = 2,055)	Model 2 (Co-Twin Report of Victimization; n = 1,985)	Model 3 (Parent Report of Victimization; n = 1,676)
Suicidal ideation	2.40 (2.11–2.74)	2.20 (1.86–2.59)	2.10 (1.73–2.56)
Self-harm	2.38 (2.10–2.69)	1.99 (1.68–2.36)	2.07 (1.71–2.50)
Suicide attempt	3.14 (2.54–3.88)	2.73 (2.21–3.39)	2.08 (1.54–2.79)

Note: Results are presented as odds ratio (95% CI).

### Are Victimized Adolescents at Greater Risk for Self-Injurious Thoughts and Behaviors Because of Confounding by Family-wide Characteristics?

Next, we examined the mechanisms underlying these associations. Adolescents experience victimization^6^ and develop self-injurious thoughts and behaviors partly because of family-wide characteristics, such as genetic vulnerabilities and the rearing environment (Figure S1, available online). Therefore, family-wide characteristics are plausible non-causal mechanisms underlying the observed associations. We tested the role of these family-wide characteristics by examining the association between adolescent victimization and self-injurious thoughts and behaviors within twin pairs who shared their rearing environment and half (dizygotic twins) or all (monozygotic twins) of their segregating genes. Twins exposed to more victimization types were at greater risk for suicidal ideation, self-harm, and suicide attempts compared with their co-twin exposed to fewer victimization types (yellow points in Figure 2; Table S5, Panel B, available online), although these effect sizes were smaller than phenotypic associations in the overall sample. Then, we restricted the analysis to genetically identical monozygotic twin pairs to fully account for confounding by genetic vulnerabilities. Within monozygotic twin pairs, adolescents exposed to more victimization types were at greater risk for suicidal ideation and self-harm, but not for suicide attempt, compared with their co-twin exposed to fewer victimization types (red points in Figure 2; Table S5, Panel D, available online). This suggests that adolescent victimization has a small unique environmental effect on suicidal ideation and self-harm. However, victimized adolescents are likely to attempt suicide because of family-wide characteristics, such as genetic vulnerability and unsupportive rearing environments.

### Are Victimized Adolescents at Greater Risk for Self-Injurious Thoughts and Behaviors Because of Confounding by Individual Factors?

**Figure 3:**
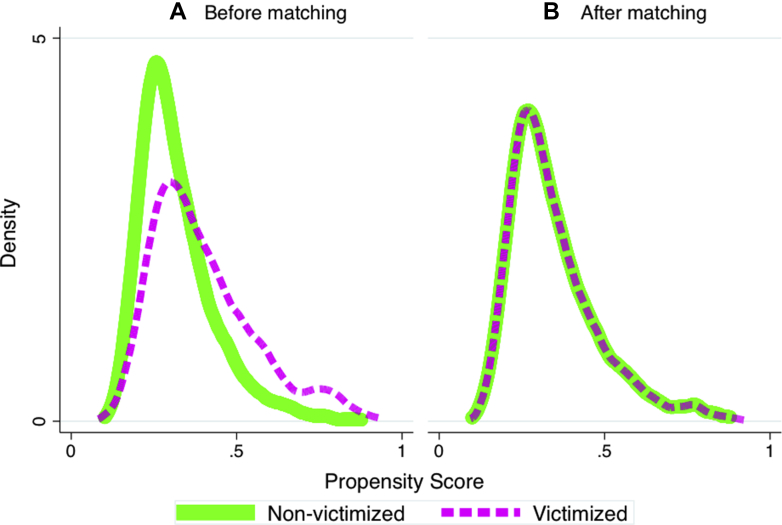
Propensity Score for Adolescent Victimization in Non-Victimized and Victimized Adolescents Based on Child-Specific Characteristics ***Note****: The propensity score was derived based on the following child-specific characteristics: childhood victimization, social isolation, IQ, internalizing problems, externalizing problems, self-harm, openness to experience, conscientiousness, extraversion, agreeableness, and neuroticism. We used 1:1 nearest neighbor matching with replacement to match each study member to a study member with a similar propensity score in the opposite “treatment” group (eg, victimization [n = 671] or no victimization [n = 1,265]). Please note color figures are available online.*

Although the co-twin control design accounts for family-wide characteristics, it cannot account for experiences or characteristics that differ for children within a family (ie, individual factors). Victimized and non-victimized adolescents differed on several pre-existing individual factors (Figure 3A; Tables S6 and S7, available online), which also predicted self-injurious thoughts and behaviors (Table S8, available online) and thus were plausible non-causal mechanisms underlying the observed associations. We tested the role of these individual factors in 3 ways.

First, we tested whether any of the selected individual risk factors could explain the association between adolescent victimization and self-injurious thoughts and behaviors. We did not find evidence of confounding by any single individual risk factor (Table S9, available online).

Second, we re-estimated the associations after matching victimized adolescents to non-victimized adolescents with a similar propensity for adolescent victimization based on 11 individual risk factors. Of note, matched victimized and non-victimized adolescents did not differ according to pre-existing individual vulnerabilities (Figure 3B; Table S6, Panel B, available online). We found that victimized adolescents showed greater risk for suicidal ideation (average treatment effect [ATE] 17.15%, 95% CI 13.16%–21.14%), self-harm (ATE 19.73%, 95% CI 15.33%–24.14%), and suicide attempt (ATE 8.06%, 95% CI 5.43%–10.68%) than matched non-victimized adolescents, although risk was on average 10% lower than in the original, non-matched analyses (Table S10, available online).

Third, to estimate the joint bias owing to family-wide and individual factors, we expanded the co-twin control analysis to include the above propensity score. Even when accounting for within-pair differences in individual characteristics, monozygotic twins exposed to more victimization types were at greater risk for suicidal ideation and self-harm than their co-twins exposed to fewer victimization types (blue points in Figure 2; Table S5, Panel E, available online).

## Discussion

We found that victimized adolescents were more likely to engage in self-injurious thoughts and behaviors than their non-victimized peers, consistent with previous research.^12^, ^13^, ^14^, ^15^ This risk was marked—exposure to each additional victimization type doubled the odds of suicidal ideation and self-harm and tripled the odds of attempting suicide—and was consistent across different informants and victimization types. Therefore, adolescent victimization is an important risk indicator for self-injurious thoughts and behaviors in young people.

To better understand the contribution of non-causal mechanisms to this association and thus inform intervention development, we used a co-twin control design to account for pre-existing family vulnerabilities and propensity score methods to account for pre-existing individual vulnerabilities. Taken together, our results both strengthen the evidence for high risk of self-injurious thoughts and behaviors in victimized adolescents and challenge conventional interpretations. Even in the most stringent analyses (the monozygotic co-twin control design accounting for individual propensity to victimization), victimized adolescents showed increased risk for suicidal ideation and self-harm, consistent with likely causal effects of adolescent victimization on psychopathology.^34^ However, these analyses also highlighted the role of pre-existing familial and individual vulnerabilities in the association, because effect sizes were substantially smaller than in the unadjusted analyses (Figure 2). This suggests that previous studies might have overestimated the causal association between adolescent victimization and self-injurious thoughts and behaviors.

Our study has limitations. First, assessment of victimization and self-injurious thoughts and behaviors spanned the same observational period and, therefore, the direction of effects is unclear. However, the findings were independent of childhood self-harm and thus are unlikely to be explained by continuity in self-injury. Second, adolescent victimization and self-injurious thoughts and behaviors were measured by self-report, potentially giving rise to common-method bias.^31^ Nevertheless, adolescent victimization remained associated with self-injurious thoughts and behaviors when victimization was reported by co-informants. Third, the effect estimates were less precise for suicide attempt because it is rarer than suicidal ideation and self-harm. Therefore, as the effect sizes were similar to those observed for other outcomes, the non-significant association between victimization and suicide attempts in monozygotic twin analyses might reflect low statistical power. Fourth, findings in our twin sample might not generalize to singletons. However, the prevalence estimates for victimization and self-injurious thoughts and behaviors reported in the present study are similar to estimates in singleton samples.^34^ Despite these limitations, our findings have implications for research and interventions.

For future research, our findings suggest the need to better understand the mechanisms linking adolescent victimization to self-injurious thoughts and behaviors. The experience of victimization might directly evoke negative self-views and, in turn, trigger suicidal ideation and self-harm as a means of escaping negative feelings or punishing oneself.^35^ Furthermore, future research should identify pre-existing familial and individual vulnerabilities that contribute to the increased risk of self-injurious thoughts and behaviors in victimized adolescents. These vulnerabilities might include partly heritable individual traits, such as poor emotion regulation, impulsivity, and low self-esteem,^36^, ^37^ as well as unsupportive family environments.^38^, ^39^

For interventions, our findings suggest that primary prevention of adolescent victimization and targeted therapeutic interventions could partly lower the risk for suicidal ideation and self-harm. Furthermore, secondary preventative strategies addressing pre-existing vulnerabilities to self-injurious thoughts and behaviors in victimized adolescents could substantially lower the risk for premature death. For example, our findings lend support to the idea that victimized adolescents are likely to benefit from phase-based approaches that include strategies to regulate arousal and negative emotions (eg, relaxation techniques)^40^ and to promote supportive family environments^38^, ^39^ before exposure and/or cognitive restructuring work to target traumatic victimization experiences.^41^
