# Supplementary material for: Adolescent Victimization and Self-Injurious Thoughts and Behaviors: A Genetically Sensitive Cohort Study
Source: J Am Acad Child Adolesc Psychiatry. 2019 May;58(5):506–13. doi: 10.1016/j.jaac.2018.07.903 (PMC6494951; doi:10.1016/j.jaac.2018.07.903)
Supplement: Supplemental Material [file mmc1.docx]

**Supplementary Material**

**Supplement 1**

*Assessment of victimization in adolescence*

We have previously reported evidence on the reliability and validity of our measurement of adolescent victimization.^1^ Here we summarize the assessment methods.

Each twin was interviewed separately at age 18 by a different research worker and was assured of the confidentiality of their responses. The participants were advised that confidentiality would only be broken if they told the research worker that they were in immediate danger of being hurt, and in such situations the project leader would be informed and would contact the participant to discuss a plan for safety.

Assessment of victimization in adolescence was based on an adapted version of the Juvenile Victimization Questionnaire 2nd revision (JVQ-R2) comprised of 5 questions that asked about maltreatment, 5 about neglect, 7 about sexual victimization, 6 about family violence, 10 about peer/sibling victimization, 3 about cyber-victimization, and 9 about crime victimization. Each JVQ-R2 question was asked for the period ‘since you were 12’. Participants were given the option to say “yes” or “no” as to whether each type of victimization had occurred in the reporting period. Research workers could rate each item “maybe” if the participant seemed unsure or hesitant in their response or they were not convinced that the participant understood the question or was paying attention. Items rated as “maybe” were recoded as “no” or “yes” by the rating team based on the notes provided by the research workers. When insufficient notes were available, these responses were recoded conservatively as a “no”. Consistent with the JVQ manual ^2,3^ participants were coded as 1 if they reported any experience within each type of victimization category, or 0 if none of the experiences within the category were endorsed. If an experience was endorsed within a victimization category, follow-up questions were asked concerning how old the participant was when it (first) happened, whether the participant was physically injured in the event, whether the participant was upset or distressed by the event; and how long it went on for (by marking the number of years on a Life History Calendar.)^4^ In addition, the interviewer wrote detailed notes based on the participant’s description of the worst event. If multiple experiences were endorsed within a victimization category, the participant was asked to identify and report about their worst experience.

All information from the JVQ interview was compiled into victimization dossiers. Using these dossiers, each of the seven victimization categories was rated by an expert in victimology and 3 other members of the E-Risk team who were trained on using the rating criteria. Ratings were made using a 6-point scale: 0 = not exposed, then 1-5 for increasing levels of severity. The anchor points for these ratings were adapted from the coding system used for the Childhood Experience of Care and Abuse interview (CECA),^5,6^ which has good inter-rater reliability.^6,7^ The CECA is a comprehensive semi-structured interview whose standardized coding system attempts to improve the objectivity of ratings by basing them on the coder’s perspective (rather than relying on the participant’s judgment) and focusing on concrete descriptions rather than perceptions or emotional responses to the questions, together with considering the context in which the adverse experience occurred.

In our adapted coding scheme, the anchor points of the scale differ for each victimization category, with some focused more on the severity of physical injury that is likely to have been incurred during victimization exposure (crime victimization, family violence, maltreatment), while others are more focused on the frequency of occurrence of victimization (peer/sibling victimization and cyber-victimization), the physical intrusiveness of the event (sexual victimization), or the pervasiveness of the effects of victimization (neglect). This reflects the different ways in which severity has previously been defined for different types of victimization.^6,8^ (Given that our sample comprises twins, we also coded if any of the victimization events experienced by each twin had been perpetrated by their co-twin, as it is possible that growing up with a genetically related, same-age child could increase or decrease sibling victimization rates.) Each twin’s dossier was evaluated separately and we did not use information provided in the co-twin’s dossier about their own or shared victimization experiences to rate direct or witnessed violence exposure for the target twin. High levels of inter-rater reliability were achieved for the severity ratings for all forms of victimization: crime victimization (intra-class correlation coefficient [ICC] = 0.89, p < 0.001), peer/sibling victimization (ICC = 0.91, p < 0.001), cyber-victimization (ICC = 0.90, p < 0.001), sexual victimization (ICC = 0.87, p < 0.001), family violence (ICC = 0.93, p < 0.001), maltreatment (ICC = 0.90, p < 0.001), and neglect (ICC = 0.74, p < 0.001).

The ratings for each type of victimization were then grouped into three classes: 0 – no exposure (score of 0), 1 – some exposure (score of 1, 2 or 3), and 2 – severe exposure (score of 4 or 5) due to small numbers for some of the rating points. Combining ratings of 4 and 5 is also consistent with previous studies using the CECA, which have collapsed comparable scale values to indicate presence of “severe” abuse. ^6,7,9,10^

The adolescent poly-victimization variable was derived by summing all victimization experiences that received a code of “2”: (i.e., severe exposure): 64.6% of adolescents had zero severe victimization experiences; 19.2% had 1; 9.4% had 2; 4.5% had 3; 1.5% had 4; 0.5.% had 5; and 0.2% had 6 severe victimization experiences. We winsorized the adolescent poly-victimization distribution into a four category variable (0, 1, 2, and 3+ severe experiences).

**Supplement 2**

*The classical twin method*

We used the classical twin method to quantify the extent to which individual differences in self-injurious thoughts and behaviors can be attributed to genetic and environmental influences, including additive genetic influences (A), shared environmental influences (C; environmental effects common to both twins), and nonshared environmental influences (E; environmental effects unique to each twin). It is possible to partition the variance in a trait in this way by exploiting the difference in genetic relatedness of monozygotic and dizygotic twin pairs. Monozygotic twins share 100% of their segregating genes, whereas dizygotic twins share on average 50% of their segregating genes. In contrast, both monozygotic and dizygotic twin pairs grow up in the same family home, and thus experience the same amount of environmental similarity, or common environment (the equal environments assumption). Therefore, genotype is the only factor that makes monozygotic twins more similar than dizygotic twins. Because of this, if monozygotic twins are more similar for self-injurious thoughts and behaviors than dizygotic twins, it implies the presence of genetic influences (A). If dizygotic twins are exactly half as similar for self-injurious thoughts and behaviors than monozygotic twins, the difference can be accounted for by their 50% difference in genetic relatedness. If dizygotic twins are more than half as similar for self-injurious thoughts and behaviors than monozygotic twins, shared environmental influences (C) are implicated. Because nonshared environmental influences make twins from the same family different, any differences in self-injurious thoughts and behaviors between monozygotic twins (who are genetically identical and experience the same common environment) must be attributed to the nonshared environment (E). E also includes measurement error.

We used the ‘OpenMx’ package in R to partition variance in self-injurious thoughts and behaviors into latent A, C, and E factors via structural equation modelling. This package estimates model parameters using maximum likelihood estimation. Because self-injurious thoughts and behaviors are categorical variables, we used liability threshold models to decompose their variances into A, C, and E factors. Liability threshold models represent self-injurious thoughts and behaviors as normally distributed latent response variables underlying the categorical outcome.^11^

The co-twin control design

We used a Generalized Estimating Equation (GEE) logistic regression model to estimate unique (within-twin pair) and family-wide (between-twin pair) effects of adolescent victimization on self-injurious thoughts and behaviors.^12-15^ We estimated the model using the following equation:

E(Y_ij_) = β_0_+ β_w_(X_ij_ - $\bar{X}$_i_) + β_B_$\bar{X}$_i_

where *i* is used to index twin pairs and *j* represents individual twins within pairs, so *E*(*Y_ij_*) represents the log(odds) for self-injurious thoughts and behaviors; *X_ij_* represents the adolescent poly-victimization score for the *j*^th^ twin of the *i*^th^ pair; and $\bar{X}$*_i_* represents the mean adolescent poly-victimization score for both twins within the *i*^th^ pair. The within-twin pair regression coefficient (β_w_) estimates whether the twin exposed to a greater number of victimization types has greater risk of self-injurious thoughts and behaviors than their less victimized co-twin. The between-twin pair coefficient (β_B_) estimates whether twin pairs exposed to a greater number of victimization types (on average) have greater risk of self-injurious thoughts and behaviors than other (less victimized) twin pairs. In the results, we report estimates of the within-twin pair regression coefficient (β_w_). We do not report between-twin pair coefficients in the analyses as our interest was in the within-pair coefficients which account for familial influences.

To account for child-specific factors influencing risk of adolescent victimization within the co-twin control design, we expanded the regression model to include individual propensity scores (see below), using the following equation:

E(Y_ij_) = β_0_+ β_1w_(X_ij_ - $\bar{X}$_i_) + β_1B_$\bar{X}$_i_ + β_2w_(X_ij_ - $\bar{X}$_i_) + β_2B_$\bar{X}$_i_

where β_1w_ and β_1B_ represent the within-twin pair regression coefficient and the between-twin pair coefficient for adolescent victimization, respectively. In addition, β_2w_ and β_2B_ represent the within-twin pair regression coefficient and the between-twin pair coefficient for propensity to adolescent victimization conditional on child-specific characteristics, respectively. Of note, in this expanded co-twin control model, the within-twin pair regression coefficient β_1w_ reported in the results estimates whether the twin exposed to a greater number of victimization types has greater risk of self-injurious thoughts and behaviors than their less victimized co-twin once within-twin pair and between-pair differences in propensity to adolescent victimization conditional on child-specific characteristics are accounted for.

**Table S1**. Description of the Child-Specific Characteristics Included in the Propensity Score for Adolescent Victimization

| **Measure** | **Age(s) assessed** | **Informant** | **Description** | **Reference** |
| --- | --- | --- | --- | --- |
| Childhood victimization | 5, 7, 10, 12 | Mother, observer, participant | Children’s exposure to domestic violence, frequent bullying by peers, physical maltreatment, sexual abuse, emotional abuse and neglect, and physical neglect was assessed. Mothers reported on their children’s exposure to domestic violence, frequent bullying by peers, physical maltreatment, sexual abuse, and emotional abuse and neglect. Research workers reported on children’s physical maltreatment, sexual abuse, emotional abuse and neglect and physical neglect (after interviewing mothers and children and observing the home-environment). Children reported on their exposure to bullying victimization at the age 12 assessment. The measure indexed the number of types of severe victimization that a child experienced. | ^21,22^ |
| Childhood social isolation | 5, 7, 10, 12 | Mother, teacher | Sum of 6-items on the CBCL and matching items on the TRF. Scores were averaged across raters at each assessment. Social isolation defined as moderately isolated at two or more ages or highly isolated at one or more ages. | ^23^ |
| Childhood IQ | 5 | Participant | Vocabulary and Block design subtests on a short form of the Wechsler Preschool and Primary Scale of Intelligence-Revised. | ^24^ |
| Childhood internalizing problems | 5, 7, 10, 12 | Mother, teacher | Sum of items on withdrawn/depressed and somatic subscales on the Child Behavior Checklist (CBCL) and teacher’s report form (TRF). Total scores standardized and averaged across raters, and assessments. | ^25,26^ |
| Childhood externalizing problems | 5, 7, 10, 12 | Mother, teacher | Sum of items on delinquency and aggression subscales on the CBCL and TRF. Total scores standardized and averaged across raters, and assessments. | ^25,26^ |
| Childhood self-harm | 10, 12 | Mother | Report of self-harm or suicide attempt made in past 6 months, at either assessment. | ^27^ |
| Openness to experience | 10 | Observer | Using a child version of the Big Five inventory, the child was rated on the following traits: perceptive, knowledgeable, original, curious, and imaginative. | ^28^ |
| Conscientiousness | 10 | Observer | Using a child version of the Big Five inventory, the child was rated on the following traits: conscientious, diligent, planful, disorderly (reverse scored), focussed, and persevering. | ^28^ |
| Extraversion | 10 | Observer | Using a child version of the Big Five inventory, the child was rated on the following traits: gregarious, energetic, cheerful, talkative, constrained (reverse scored), and merry. | ^28^ |
| Agreeableness | 10 | Observer | Using a child version of the Big Five inventory, the child was rated on the following traits: considerate, rude (reverse scored), trusting, spiteful (reverse scored), angry (reverse scored). | ^28^ |
| Neuroticism | 10 | Observer | Using a child version of the Big Five inventory, the child was rated on the following traits: touchy, brave (reverse scored), tense, tearful, fearful. | ^28^ |

**Table S2.** Characteristics of Study Members with Missing Data for the Propensity Score based on Child-Specific Variables

| Variable | Level | Study members with complete data for the propensity score (n=1936) | Study members missing data for the propensity score (n=119) | p-value |
| --- | --- | --- | --- | --- |
| *Adolescent victimization* | *0 types* | 1265 (65.34) | 62 (52.10) | 0.32 |
|  | *1 type* | 365 (18.85) | 31 (26.05) |  |
|  | *2 types* | 180 (9.30) | 14 (11.76) |  |
|  | *3+ types* | 126 (6.51) | 12 (10.08) |  |
| *Suicidal ideation* |  | 250 (12.91) | 21 (17.65) | 0.13 |
| *Self-harm* |  | 260 (13.43) | 15 (12.61) | 0.38 |
| *Suicide attempt* |  | 73 (3.77) | 6 (5.04) | 0.63 |

Note: Results are presented as no. (%). *p*-values were estimated from GEE logistic regression models accounting for familial clustering.

**Table S3**. Tests of Sex Differences Between Adolescent Poly-victimization and Self-injurious Thoughts and Behaviors

| Type | Suicidal ideation | Self-harm | Suicide attempt |
| --- | --- | --- | --- |
| *Poly-victimization* | 2.23 (1.42-3.50) | 2.15 (1.41-3.26) | 3.94 (2.10-7.39) |
| *Gender* | 1.15 (0.77-1.72) | 1.64 (1.09-2.46) | 1.47 (0.60-3.59) |
| *Gender*poly-victimization* | 1.05 (0.80-1.37) | 1.06 (0.83-1.36) | 0.87 (0.58-1.30) |

Note: Results are presented as Odds Ratios and 95% confidence intervals.

**Table S4**. Associations Between Types of Adolescent Victimization and Self-injurious Thoughts and Behaviors.

| Victimization type | Suicidal ideation | Self-harm | Suicide attempt |
| --- | --- | --- | --- |
| *Maltreatment (n=67)* | 11.70 (6.80-20.11) | 8.27 (5.11-13.39) | 8.69 (4.55-16.61) |
| *Neglect (n=46)* | 10.50 (5.80-19.00) | 9.43 (5.25-16.95) | 8.70 (3.98-19.02) |
| *Family violence (n=249)* | 3.20 (2.31-4.42) | 3.19 (2.29-4.43) | 3.84 (2.29-6.44) |
| *Sexual victimization (n=53)* | 10.36 (5.66-18.97) | 18.48 (10.61-32.18) | 10.42 (4.87-22.26) |
| *Peer/sibling victimization (n=322)* | 3.51 (2.62-4.71) | 3.60 (2.69-4.82) | 5.65 (3.56-8.97) |
| *Cyber-victimization (n=132)* | 2.67 (1.74-4.10) | 2.45 (1.59-3.77) | 4.33 (2.36-7.93) |
| *Crime victimization (n=396)* | 3.68 (2.75-4.93) | 3.40 (2.57-4.49) | 8.26 (4.91-13.91) |

Note: Results are presented as Odds Ratios and 95% confidence intervals. The n reported is the number of Study members who reported each victimization type.

**Table S5**. Association Between Adolescent Victimization and Self-injurious Thoughts and Behaviors Within Twin-pairs

|  | Panel A  Phenotypic association  (N=2055) | Panel B  MZ and DZ twin pairs  (n^pairs^=1043) | Panel C  DZ twin pairs  (n^pairs^=458) | Panel D  MZ twin pairs  (n^pairs^=550)^a^ | Panel E  MZ twin pairs and propensity score  (n^pairs^=550)^a^ |
| --- | --- | --- | --- | --- | --- |
| *Suicidal ideation* | 2.40 (2.11-2.74) | 1.73 (1.41-2.13) | 2.07 (1.51-2.84) | 1.46 (1.11-1.93) | 1.45 (1.10-1.91) |
| *Self-harm* | 2.38 (2.10-2.69) | 1.82 (1.50-2.22) | 2.39 (1.72-3.32) | 1.51 (1.18-1.92) | 1.50 (1.18-1.91) |
| *Suicide attempt* | 3.14 (2.54-3.88) | 2.02 (1.39-2.92) | 3.12 (1.94-5.03) | 1.31 (0.85-2.00) | 1.28 (0.83-1.98) |

Note: The phenotypic associations are the associations between adolescent victimization and self-injurious thoughts and behaviors treating each twin as an individual (accounting for familial clustering), and the other columns show the associations between within-twin pair differences in victimization and self-injurious thoughts and behaviors. Results are presented as Odds Ratios with 95% confidence intervals.

DZ = dizygotic. MZ = monozygotic.

^a^The sample size for the within-MZ twin pair analyses (Panels D and E) was restricted to MZ twin pairs with complete data for the propensity score (n^pairs^=550), to ensure consistency in sample size across analyses that did or did not control for the propensity score. Of note, findings in Panel D were similar when the unrestricted sample of MZ twin pairs (n^pairs^=1043-458=585) was used instead: adolescent victimization was associated with suicidal ideation (OR=1.43, 95% CI=1.10-1.87) and self-harm (OR=1.47, 95% CI=1.17-1.86), but not suicide attempt (OR=1.36, 95% CI=0.88-2.12).

**Table S6**. Distribution of Child-specific Covariates in Victimized and Non-victimized Adolescents, Before and After Matching by Propensity Score.

| Covariate | Panel A: Before matching by propensity score | | | Panel B: After matching by propensity score | | |
| --- | --- | --- | --- | --- | --- | --- |
|  | **Victimized adolescents (n=671)** | **Non-victimized adolescents (n=1265)** | **Standardized difference** | **Victimized adolescents (n=671)** | **Non-victimized adolescents (n=1265)** | **Standardized difference** |
| *Child victimization* | 0.56 | 0.27 | 0.38 | 0.56 | 0.53 | 0.04 |
| *Child social isolation* | 0.43 | 0.28 | 0.30 | 0.43 | 0.43 | 0.00 |
| *Child IQ* | 93.94 | 97.08 | -0.22 | 93.94 | 93.64 | -0.04 |
| *Child self-harm* | 0.06 | 0.03 | 0.15 | 0.06 | 0.05 | 0.01 |
| *Child internalizing problems* | 12.47 | 10.93 | 0.24 | 12.47 | 12.55 | 0.01 |
| *Child externalizing problems* | 19.90 | 14.40 | 0.46 | 19.90 | 19.90 | 0.00 |
| *Openness to experience* | 5.80 | 5.91 | -0.04 | 5.80 | 5.84 | -0.09 |
| *Conscientiousness* | 8.37 | 8.91 | -0.17 | 8.37 | 8.31 | -0.06 |
| *Extraversion* | 9.05 | 8.66 | 0.12 | 9.05 | 9.05 | -0.05 |
| *Agreeableness* | 8.29 | 8.75 | -0.24 | 8.29 | 8.23 | -0.01 |
| *Neuroticism* | 1.94 | 1.91 | 0.02 | 1.94 | 2.01 | 0.01 |

Note: The table shows means and standardized differences between victimized and non-victimized participants, before and after matching with replacement by the propensity score. A standardized difference of over 0.10 indicates covariate imbalance between groups.^17^ Before matching, the standardized differences across the 11 covariates ranged from a minimum of 0.02 (neuroticism) to a maximum of 0.46 (childhood externalizing problems) with a mean of 0.21, indicating that victimized and non-victimized adolescents differed in these child-specific factors. After matching by propensity score, the standardized differences ranged from a minimum of 0.00 (childhood social isolation; childhood externalizing problems) to a maximum of 0.09 (openness to experience) with a mean of 0.03, indicating that the covariates were balanced between victimized and non-victimized adolescents. We did not use statistical significance testing to compare the background covariates among victimized and non-victimized participants matched for propensity scores because variation in sample size in the matched sample could affect significance values.^18-20^

**Table S7.** Association Between Child-specific Covariates Included in the Propensity Score with Adolescent Victimization.

| Covariate | Adolescent victimization | | p-value |
| --- | --- | --- | --- |
|  | **Victimized**  **(n=671)** | **Non-victimized (n=1265)** |  |
| *Child victimization* | 0.56 (0.87) | 0.27 (0.60) | <0.001 |
| *Child social isolation, N (%)* | 286 (42.62) | 357 (28.22) | <0.001 |
| *Child IQ* | 93.94 (14.22) | 97.08 (14.49) | <0.001 |
| *Child self-harm, N (%)* | 38 (5.66) | 34 (2.69) | 0.049 |
| *Child internalizing problems* | 12.47 (7.04) | 10.93 (5.98) | 0.001 |
| *Child externalizing problems* | 19.89 (13.33) | 14.40 (10.53) | <0.001 |
| *Openness to experience* | 5.80 (2.78) | 5.91 (2.85) | 0.29 |
| *Conscientiousness* | 8.37 (3.29) | 8.91 (3.12) | 0.003 |
| *Extraversion* | 9.04 (3.25) | 8.66 (3.30) | 0.036 |
| *Agreeableness* | 8.29 (2.15) | 8.75 (1.70) | <0.001 |
| *Neuroticism* | 1.94 (1.89) | 1.91 (1.90) | 0.86 |

Note: Results are presented as means (and standard deviations) unless otherwise specified. P-values were derived from GEE logistic regression models accounting for familial clustering.

**Table S8.** Association Between Child-specific Covariates Included in the Propensity Score with Adolescent Self-injurious Thoughts and Behaviours.

| Covariate | Suicidal ideation | Self-harm | Suicide attempt |
| --- | --- | --- | --- |
| *Child victimization* | 1.67 (1.42-1.97) | 1.60 (1.35-1.90) | 1.78 (1.44-2.21) |
| *Child social isolation* | 2.45 (1.88-3.21) | 1.89 (1.45-2.48) | 3.03 (1.88-4.89) |
| *Child IQ* | 0.99 (0.98-0.99) | 0.99 (0.98-1.00) | 0.99 (0.97-1.00) |
| *Child self-harm* | 3.62 (2.16-6.06) | 3.39 (2.00-5.74) | 5.63 (2.83-11.31) |
| *Child internalizing problems* | 1.06 (1.04-1.08) | 1.06 (1.04-1.08) | 1.07 (1.04-1.10) |
| *Child externalizing problems* | 1.03 (1.02-1.04) | 1.02 (1.01-1.03) | 1.03 (1.01-1.05) |
| *Openness to experience* | 1.03 (0.98-1.08) | 1.02 (0.97-1.07) | 1.00 (0.91-1.11) |
| *Conscientiousness* | 0.96 (0.92-1.00) | 0.95 (0.91-0.99) | 0.88 (0.83-0.94) |
| *Extraversion* | 0.98 (0.94-1.03) | 1.02 (0.98-1.07) | 1.01 (0.93-1.09) |
| *Agreeableness* | 0.88 (0.82-0.94) | 0.93 (0.87-1.00) | 0.82 (0.74-0.91) |
| *Neuroticism* | 1.08 (1.01-1.16) | 1.05 (0.99-1.13) | 1.11 (0.99-1.25) |

Note: Odds ratios (and 95% confidence intervals) were estimated from GEE logistic regression models accounting for familial clustering.

**Table S9.** Association Between Adolescent Victimization and Self-injurious Thoughts and Behaviors After Adjusting for Each Child-specific Covariate Included in the Propensity Score.

| Covariate adjusted for | Suicidal ideation | Self-harm | Suicide attempt |
| --- | --- | --- | --- |
| *Unadjusted* | 2.40 (2.11-2.74) | 2.38 (2.10-2.69) | 3.14 (2.54-3.88) |
| *Child victimization* | 2.25 (1.97-2.57) | 2.29 (2.01-2.60) | 2.94 (2.33-3.72) |
| *Child social isolation* | 2.29 (2.00-2.61) | 2.32 (2.05-2.63) | 2.97 (2.38-3.71) |
| *Child IQ* | 2.34 (2.05-2.67) | 2.37 (2.09-2.69) | 3.06 (2.46-3.82) |
| *Child self-harm* | 2.33 (2.04-2.66) | 2.35 (2.07-2.66) | 3.00 (2.41-3.74) |
| *Child internalizing problems* | 2.31 (2.02-2.64) | 2.33 (2.05-2.64) | 3.00 (2.40-3.74) |
| *Child externalizing problems* | 2.29 (2.00-2.62) | 2.34 (2.06-2.67) | 3.01 (2.39-3.80) |
| *Openness to experience* | 2.36 (2.07-2.70) | 2.38 (2.10-2.69) | 3.10 (2.49-3.86) |
| *Conscientiousness* | 2.36 (2.06-2.70) | 2.37 (2.09-2.69) | 3.08 (2.45-3.89) |
| *Extraversion* | 2.40 (2.10-2.74) | 2.38 (2.10-2.70) | 3.13 (2.53-3.87) |
| *Agreeableness* | 2.34 (2.04-2.67) | 2.37 (2.09-2.69) | 3.04 (2.43-3.82) |
| *Neuroticism* | 2.37 (2.07-2.72) | 2.38 (2.10-2.70) | 3.09 (2.48-3.85) |

Note: Odds ratios (and 95% confidence intervals) were estimated from GEE logistic regression models accounting for familial clustering.

**Table S10.** Difference in Percentage of Self-injurious Thoughts and Behaviors Between Victimized and Non-victimized Adolescents, Before and After Matching by the Propensity Score.

|  | Suicidal ideation | Self-harm | Suicide attempt |
| --- | --- | --- | --- |
| *% difference in prevalence in unmatched analyses (95% CI) ^1^* | 19.24 (16.22;22.26) | 21.87 (18.83;24.91) | 9.05 (7.31;10.79) |
| *% difference in prevalence in matched analyses (ATE)(95% CI) ^2^* | 17.15 (13.16;21.14) | 19.73 (15.33;24.14) | 8.06 (5.43;10.68) |
| *% difference between unmatched and matched analyses (95% CI)* | -10.86 (-11.11;-10.61) | -9.79 (-10.03;-9.55) | -10.94 (-11.28;-10.60) |

Note: *^1^* The percentage difference of self-injurious thoughts and behaviors between victimized and non-victimized adolescents before matching by the propensity score was derived by i) tabulating self-injurious thoughts and behaviors by victimization, and ii) calculating the difference in percentages of self-injurious thoughts and behaviors between victimized and non-victimized adolescents. Positive values indicate that the percentage of adolescents with the outcome is higher in victimized relative to non-victimized adolescents.

*^2^* The expected percentage difference of self-injurious thoughts and behaviors between victimized and non-victimized adolescents after matching by the propensity score is the average treatment effect (ATE), calculated using the command ‘teffects psmatch’ in Stata. Positive values indicate that the percentage of adolescents with the outcome is higher in victimized relative to non-victimized adolescent

**Figure S1**. Genetic and Environmental Influences on Self-injurious Thoughts and Behaviors.

Note: The barplot shows the percentage of variance in self-injurious thoughts and behaviors accounted by additive genetic influences (A) and unique environmental influences (E), as the shared environment (C) explained 0% of the variance in these phenotypes. The cross-twin pair tetrachoric correlations for suicidal ideation were r=0.60 (monozygotic twins) and r=0.32 (dizygotic twins); the correlations for self-harm were r=0.60 (monozygotic twins) and r=0.16 (dizygotic twins); and the correlations for suicide attempt were r=0.63 (monozygotic twins) and r=0.25 (dizygotic twins). The additive genetic influence on self-harm and suicide attempt was 0%.

**Supplementary References**

**1.** Fisher HL, Caspi A, Moffitt TE, et al. Measuring adolescents' exposure to victimization: The Environmental Risk (E-Risk) Longitudinal Twin Study. *Dev. Psychopathol.* 2015;27(4pt2):1399-1416.

**2.** Finkelhor D, Hamby S, Turner H, Ormod R. *The Juvenile Victimization Questionnaire: 2nd Revision (JVQ-R2).* Durham, NH: Crimes Against Children Research Center; 2011.

**3.** Hamby S, Finkelhor D, Ormrod D, Turner H. *The comprehensive JVQ administration and scoring manual.* Durham, NH: University of New Hampshire: Crimes Against Children Research Centre; 2004.

**4.** Caspi A, Moffitt TE, Thornton A, et al. The life history calendar: a research and clinical assessment method for collecting retrospective event-history data. *Int. J. Methods Psychiatr. Res.* 1996.

**5.** Bifulco A, Brown G, Neubauer A, Moran P, Harris T. *Childhood Experience of Care and Abuse (CECA) training manual.* London: Royal Holloway, University of London; 1994.

**6.** Bifulco A, Brown GW, Harris TO. Childhood Experience of Care and Abuse (CECA): a retrospective interview measure. *J Child Psychol Psychiatry.* 1994;35(8):1419-1435.

**7.** Bifulco A, Brown G, Lillie A, Jarvis J. Memories of childhood neglect and abuse: corroboration in a series of sisters. *J Child Psychol Psychiatry.* 1997;38(3):365-374.

**8.** Barnett D, Manly JT, Cicchetti D. Defining child maltreatment: The interface between policy and research. In: Cicchetti D, Toth SL, eds. *Child Abuse, Child Development, and Social Policy*. Norwood, NJ: Ablex; 1993:7-73.

**9.** Bifulco A, Brown G, Moran P, Ball C, Campbell C. Predicting depression in women: the role of past and present vulnerability. *Psychol. Med.* 1998;28(1):39-50.

**10.** Fisher HL, Bunn A, Jacobs C, Moran P, Bifulco A. Concordance between mother and offspring retrospective reports of childhood adversity. *Child Abuse Neglect.* 2011;35(2):117.

**11.** Rijsdijk FV, Sham PC. Analytic approaches to twin data using structural equation models. *Brief Bioinform.* 2002;3(2):119-133.

**12.** Carlin JB, Gurrin LC, Sterne JA, Morley R, Dwyer T. Regression models for twin studies: a critical review. *Int. J. Epidemiol.* 2005;34(5):1089-1099.

**13.** Arseneault L, Milne BJ, Taylor A, et al. Being bullied as an environmentally mediated contributing factor to children's internalizing problems: a study of twins discordant for victimization. *Arch. Pediatr. Adolesc. Med.* 2008;162(2):145-150.

**14.** Rooks C, Veledar E, Goldberg J, Bremner JD, Vaccarino V. Early trauma and inflammation: role of familial factors in a study of twins. *Psychosom. Med.* 2012;74(2):146-152.

**15.** Schaefer JD, Moffitt, T.E., Arseneault, L., Danese, A., Fisher, H.L., Houts, R., Sheridan, M.A., Wertz, J., Caspi, A. Adolescent victimization and early-adult psychopathology: Approaching causal inference using a longitudinal twin study to rule out non-causal explanations. *Clin Psychol Sci.* 2017. doi.org/10.1177/2167702617741381.

**16.** Austin PC. An introduction to propensity score methods for reducing the effects of confounding in observational studies. *Multivariate Behavioral Research.* 2011;46(3):399-424.

**17.** Austin PC, Grootendorst P, Anderson GM. A comparison of the ability of different propensity score models to balance measured variables between treated and untreated subjects: a Monte Carlo study. *Stat. Med.* 2007;26(4):734-753.

**18.** Ho DE, Imai K, King G, Stuart EA. Matching as nonparametric preprocessing for reducing model dependence in parametric causal inference. *Political Analysis.* 2007;15(3):199-236.

**19.** Austin PC. A critical appraisal of propensity‐score matching in the medical literature between 1996 and 2003. *Stat. Med.* 2008;27(12):2037-2049.

**20.** Imai K, King G, Stuart EA. Misunderstandings between experimentalists and observationalists about causal inference. *J. Roy. Stat. Soc. Ser. A. (Stat. Soc.).* 2008;171(2):481-502.

**21.** Baldwin JR, Arseneault L, Caspi A, et al. Childhood victimization and inflammation in young adulthood: A genetically sensitive cohort study. *Brain. Behav. Immun.* 2018;67:211-217.

**22.** Danese A, Moffitt TE, Arseneault L, et al. The origins of cognitive deficits in victimized children: implications for neuroscientists and clinicians. *Am. J. Psychiatry.* 2017;174(4):349-361.

**23.** Matthews T, Danese A, Wertz J, et al. Social isolation and mental health at primary and secondary school entry: a longitudinal cohort study. *J. Am. Acad. Child Adolesc. Psychiatry.* 2015;54(3):225-232.

**24.** Wechsler D. *Wechsler preschool and primary scale of intelligence-revised.* London, England: Psychological Corporation; 1990.

**25.** Achenbach T. *Manual for the Child Behaviour Checklist and 1991 profile.* Burlington, VT: Department of Psychiatry, University of Vermont; 1991.

**26.** Achenbach T. *Manual for the Teacher's Report Form and 1991 Profile.* Burlington, VT: Department of Psychiatry, University of Vermont; 1991.

**27.** Fisher HL, Moffitt TE, Houts RM, Belsky DW, Arseneault L, Caspi A. Bullying victimisation and risk of self harm in early adolescence: longitudinal cohort study. *BMJ.* 2012;344:e2683.

**28.** Digman JM, Shmelyov AG. The structure of temperament and personality in Russian children. *J. Pers. Soc. Psychol.* 1996;71(2):341.
